# Investigating the effectiveness of combining high-frequency chest wall oscillation with bilevel positive airway pressure in pneumonia patients: a retrospective cohort study

**DOI:** 10.1186/s12890-025-03685-y

**Published:** 2025-05-03

**Authors:** Ta-Wei Chao, Ya-Chen Kao, Hui-Ling Liu, Sheng-Hsiang Lin, Chin-Wei Kuo

**Affiliations:** 1 Department of Respiratory Therapy, Department of Internal Medicine, National Cheng Kung University Hospital, Tainan, Taiwan; 2 Department of Internal Medicine, College of Medicine, National Cheng Kung University Hospital, National Cheng Kung University, Tainan, Taiwan; 3 Institute of Clinical Medicine, College of Medicine, National Cheng Kung University, 138 Sheng-Li Road, Tainan, 70403 Taiwan; 4 Biostatistics Consulting Center, National Cheng Kung University Hospital, College of Medicine, National Cheng Kung University, Tainan, Taiwan; 5 Department of Public Health, College of Medicine, National Cheng Kung University, Tainan, Taiwan; 6 Division of Chest Medicine, Department of Internal Medicine, College of Medicine, National Cheng Kung University Hospital, National Cheng Kung University, Tainan, Taiwan

**Keywords:** Pneumonia, High-frequency chest wall Oscillation, Intermittent positive pressure breathing, Hospital days

## Abstract

**Background:**

Pneumonia represents a significant global health burden with high morbidity and mortality rates, despite advances in therapeutic and preventive strategies. Airway clearance techniques (ACT), including High-Frequency Chest Wall Oscillation (HFCWO) and bilevel positive airway pressure (BiPAP), are critical in managing respiratory conditions. However, the combined effectiveness of BiPAP and HFCWO in treating adult pneumonia remains underexplored.

**Methods:**

A retrospective cohort study was conducted at a college hospital in southern Taiwan, enrolling patients aged ≥ 18 years, admitted for pneumonia from January 2020 to December 2022, who received HFCWO therapy for ≥ 5 days in the ordinary ward. Exclusion criteria included prior mechanical ventilation before HFCWO initiation. Univariate and multivariable logistic regression models were used to assess the effectiveness of the combined use of BiPAP and HFCWO.

**Results:**

A total of 271 patients received HFCWO and were enrolled for analysis, including 163 patients who received both BiPAP and HFCWO. Patients receiving both BiPAP and HFCWO were associated with decreased frequency of sputum suction (OR: 2.91, 95% CI: 1.46–5.78, *P* = 0.002), and reduced oxygen need post-HFCWO (OR: 0.55, 95% CI: 0.33–0.91, *P* = 0.021). However, there was no difference in hospital stay, respiratory failure, ICU admission, or hospital death between the groups. Additionally, there was no difference in these outcomes for patients who received HFCWO twice daily compared to those who received it once daily.

**Conclusions:**

Combining BiPAP and HFCWO reduces the need for sputum suction and improves oxygen demand for patients but does not change hospital days, respiratory failure, or mortality. Further large prospective cohort studies are necessary to confirm the efficacy of this management approach.

**Supplementary Information:**

The online version contains supplementary material available at 10.1186/s12890-025-03685-y.

## Introduction

Pneumonia remains a major public health concern worldwide, with significant mortality and morbidity rates, particularly among hospitalized patients. Despite advances in prevention and treatment, including antibiotics, the high incidence of pneumonia continues to impose a heavy burden on healthcare systems [1]. Although supportive therapies such as oxygen therapy and chest physiotherapy (CPT) have proven beneficial, there is increasing interest in optimizing airway clearance strategies in pneumonia patients to improve clinical outcomes [2].

Airway clearance techniques (ACT) play a crucial role in managing conditions where mucus accumulation and airway obstruction contribute to respiratory compromise. High-frequency chest wall oscillation (HFCWO), a well-established ACT, has been shown to improve secretion clearance by using an inflatable vest to generate rapid inflation and deflation, transmitting oscillations through the chest wall to mobilize mucus from the lungs [3, 4]. While HFCWO has demonstrated efficacy in conditions like cystic fibrosis, chronic obstructive pulmonary disease (COPD), and bronchiectasis [5–11], its application in pneumonia, particularly in combination with other treatments, remains underexplored. Bilevel positive airway pressure (BiPAP), which provides positive airway pressure during both the inspiratory and expiratory phases via a non-invasive interface, has been shown to provide clinical benefits for patients with acute exacerbations of COPD, heart failure-related respiratory failure, post-extubation, obesity hypoventilation syndrome, and obstructive sleep apnea [12]. Additionally, several pilot studies have demonstrated that BiPAP, when used to support ACT, can augment tidal volumes and reduce patient effort, thereby enhancing sputum clearance in patients with cystic fibrosis or severe obstructive disease [13, 14]. However, the clinical benefits of combining BiPAP and HFCWO for pneumonia treatment have yet to be fully evaluated.

In this study, we hypothesized that the combination of BiPAP and HFCWO would yield superior clinical outcomes compared to either treatment alone or no additional treatment. To test this hypothesis, we conducted a retrospective cohort study at a college hospital in southern Taiwan to investigate the clinical effectiveness of this combination therapy in pneumonia patients.

## Methods

### Patient enrollment and study design

This study was conducted at National Cheng Kung University Hospital (NCKUH), a college hospital serving a population of 1.86 million inhabitants in Tainan City, Taiwan, as of 2022. The Institutional Review Board of NCKUH approved this study before its commencement (Approval Number: A-ER-112-261). Informed consent was waived due to the retrospective study design. Patients aged ≥ 18 years old, admitted to NCKUH for pneumonia between January 1, 2020, and December 31, 2022, and receiving HFCWO therapy were enrolled. Pneumonia was defined by the following criteria: (1) a diagnosis of pneumonia in the discharge note, (2) presence of productive cough in the medical record, (3) lung infiltrates on chest X-ray, and (4) receipt of antibiotic treatment. Patients were excluded if they were (1) received HFCWO for less than 5 days, (2) received HFCWO in the ICU, (3) long-term mechanical ventilation dependence. We excluded patients who received less than 5 days of HFCWO treatment, as the standard treatment course for pneumonia with HFCWO in our hospital is a minimum of 5 days. This recommendation is based on previous studies suggesting that the duration of HFCWO typically ranges from 3 to 14 days [15]. Additionally, the minimum duration for antibiotic treatment is generally 5 days [16]. For patients who received multiple HFCWO therapy sessions during their hospitalization, only the first HFCWO therapy session was included into the analysis. To prevent repeated measurements, we only analyzed the first admission for patients with multiple admissions for pneumonia during the study period.

### Data collection, and the definition of variables and outcomes

Patient information, including age, sex, body mass index (BMI), smoking status, comorbidities, oxygen usage, frequency of sputum suction, frequency and duration of HFCWO, the use of BiPAP, chest X-ray (CXR) results, medication use for pneumonia, hospitalization duration, mechanical ventilation usage, admission to the ICU, and hospital mortality, was extracted from electronic medical records in the NCKUH database. Hospital-acquired pneumonia was defined as pneumonia occurring after 48 h of hospitalization or within 14 days following the previous hospitalization. Patients received non-invasive ventilation (NIV), invasive mechanical ventilation (IMV) or dead of pneumonia were defined as respiratory failure. The frequency of sputum suction indicates the average number of suction times during the 3 days before and after the completion of HFCWO therapy. Additionally, oxygen usage refers to the highest-intensity oxygen delivery device used during the 3 days before and after the completion of HFCWO therapy. The definition of post-HFCWO use hospital days is the interval between the last day of HFCWO use and the discharge day.

**Fig. 1:**
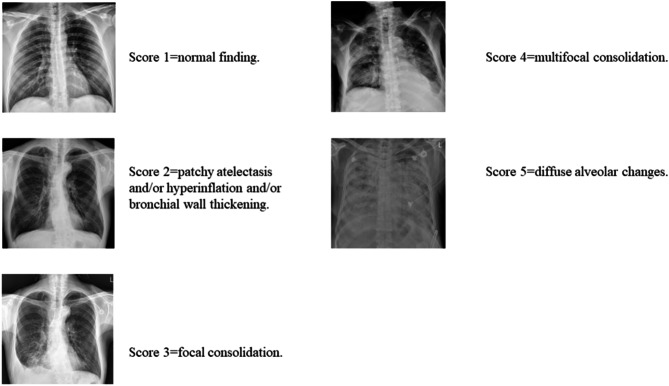
The chest X-ray findings in the Severe Acute Respiratory Infection (SARI) CXR severity scoring system

Pneumonia severity was assessed using the Severe Acute Respiratory Infection (SARI) CXR severity scoring system, a validated five-point CXR scoring tool that demonstrates good agreement among clinicians with varying levels of experience [17]. In brief, the SARI scoring system categorizes patients into five levels based on CXR findings. Patients with normal findings, patchy atelectasis and/or hyperinflation and/or bronchial wall thickening, focal consolidation, multifocal consolidation, and diffuse alveolar changes are classified as scores 1 to 5, respectively. (Fig. 1) In this study, the SARI score was initially calculated by an internal medicine doctor and subsequently verified by a pulmonologist.

### The indication and setting of HFCWO and bipap

The indications for HFCWO in pneumonia patients at NCKUH include poor airway clearance function and comorbidities such as COPD, cystic fibrosis (CF), bronchiectasis with high sputum volume, and pulmonary atelectasis. Contraindications for HFCWO include unstable hemodynamic, massive hemoptysis, increase intracranial pressure (IICP), rib fracture, chest wall injury, and recent chest surgery. BiPAP is routinely added for patients receiving HFCWO, except for those at risk of pneumothorax (e.g., large bullae, history of pneumothorax, or recent chest surgery or biopsy), unable to cooperate with BiPAP, IICP, or with facial trauma or deformity. All applications of HFCWO and BiPAP at NCKUH require approval from a pulmonologist and respiratory therapist following a comprehensive patient evaluation.

The medical device for HFCWO is COMFORTCOUGH^®^ II (Seoil Pacific Corp., South Korea). We configure the HFCWO device to “percussor” mode, setting the amplitude and frequency of percussion pressure based on patient comfort and clinical effectiveness. The amplitude of pressure was adjusted from 40 to 60 cmH_2_O and the frequency from 400 to 600 Hz, once or twice daily dependent on the availability of devices and respiratory therapists. The BiPAP device used is FlexoTM Bi-Level, set to “S/T” mode. The settings for inspiratory positive airway pressure (IPAP) and expiratory positive airway pressure (EPAP) are 8–15 cmH_2_O and 6–8 cmH_2_O, respectively. These settings aim to achieve a lung volume expansion targeted at 1.5 times the predicted tidal volume, which is calculated based on the predicted body weight (8–10 ml/kg of predicted body weight). The respiratory rate was set at 10–12 breaths per minute, with the inspiratory time set at approximately 33% of the single breathing cycle. Each setting was adjusted based on clinical effectiveness and patient comfort. BiPAP was administered contemporaneously with HFCWO, lasting about 30 min per session, once daily. For patients with significant sputum production, and when equipment and staff are available, BiPAP and HFCWO can be administered twice daily.

### Statistical analysis

The collected data were presented as numbers (percentages), means (standard deviations), or medians (interquartile range [IQR]) depending on the nature of the data. Independent t-tests and Mann-Whitney U tests were utilized to analyze continuous variables with or without a normal distribution, respectively. Fisher’s exact test was employed to analyze categorical variables. Univariable and multivariable logistic regression analyses were conducted to investigate the effectiveness of BiPAP and HFCWO. Multivariate model adjusted the covariates including the group of BiPAP use and other covariates selected in the univariate model with p-values less than 0.1. Associations were established using odds ratios (ORs) and 95% confidence intervals (CIs). All P-values were calculated as two-sided, and a *P* < 0.05 was considered statistically significant. Additionally, we performed several subgroup analyses based on oxygen demand, comorbidities (COPD, bronchiectasis), and suction frequency to further strengthen the validity of our results. All statistical analyses were carried out using SAS software (version 9.4; SAS Institute, Cary, NC, USA).

## Results

**Fig. 2:**
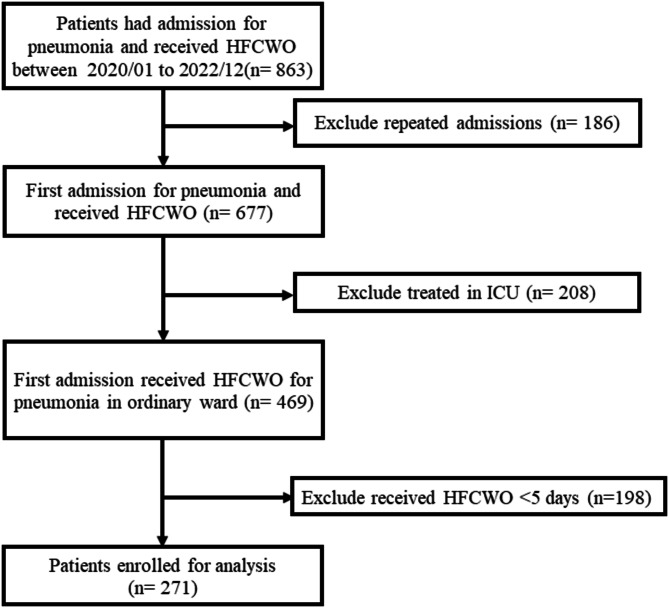
The algorithm for patient enrollment in this study

Between January 1, 2020, and December 31, 2022, a total of 863 patients at NCKUH received HFCWO for pneumonia. After excluding patients with repeated admissions for pneumonia (*n* = 186), those treated in the ICU (*n* = 208), and those who received HFCWO for less than 5 days (*n* = 198), 271 patients were eligible for analysis **(**Fig. 2**)**. Among these patients, 163 received a combination of BiPAP and HFCWO for pneumonia, while 108 received HFCWO alone **(**Table 1**)**. Compared to patients who received HFCWO alone, those who received both BiPAP and HFCWO were older, need lower oxygen support level and required more frequent sputum suctioning. There was no difference between the two groups in sex, BMI, smoking status, comorbidities, baseline SARI CXR scores, the number of HFCWO days, or the frequency of HFCWO use.

**Table 1:** Basic demographic information of enrolled patients who received HFCWO treatment

	No BiPAP use (*N* = 108)		BiPAP use (*N* = 163)		
	N or median	(%) or IQR	N or median	(%) or IQR	P-value^*^
**Basic information**					
Age, median (IQR)(year)	78.5	(70, 85.5)	83	(74, 89)	0.008
Male, n (%)	78	72.22	116	71.17	0.959
BMI level, n (%)					0.290
Underweight	30	27.8	47	28.8	
Normal	63	58.3	103	63.2	
Overweight	15	13.9	13	8.0	
Active smoking, n (%)	16	14.8	16	9.8	0.291
Comorbidities, n (%)					
COPD	19	17.6	27	16.6	0.956
Bronchiectasis	11	10.2	11	6.8	0.431
Pulmonary fibrosis	2	1.9	6	3.7	0.483^†^
CVA	30	27.8	43	26.4	0.909
Heart failure	19	17.6	39.	23.9	0.274
CKD	30	27.8	49	30.1	0.788
**Baseline clinical severity**					
Oxygen demand, n (%)					< 0.001
Room air or N/C	36	33.3	85	52.2	
VM FiO_2_ < 50%	24	22.2	43	26.4	
VM FiO_2_ ≥ 50% or MV	48	44.4	35	21.5	
Sputum suction frequency	0	(2.5, 16)	12	(3, 19)	< 0.001
Baseline SARI CXR score					0.186
>2	94	87.0	151	92.6	
≤2	14	13.0	12	7.4	
Types of pneumonia					0.078
Community-acquired	57	52.8	104	63.8	
Hospital-acquired	51	47.2	59	36.2	
Pneumonia treatment					
Antibiotics	108	100	163	100	1.000
Mucolytics	106	98.1	159	97.5	1.000
Bronchodilator	78	72.2	129	79.1	0.193
**HFCWO use**					
HFCWO days, median (IQR)	7	(6, 11)	8	(6, 10)	0.427
HFCWO frequency, n (%)					0.576
Once daily	23	21.3	29	17.8	
Twice daily	85	78.7	134	82.2	

^*^Fisher exact test and independent t test were used to calculate continuous variables and category variables, respectively

BMI, body mass index; BiPAP, Bilevel positive airway pressure; COPD, chronic obstructive pulmonary disease; CKD, chronic kidney disease; CVA, cerebrovascular accident; DM, diabetes mellitus; HFCWO, high frequency chest wall oscillation; ICU, intensive care unit; MV, mechanical ventilation; NIV, non-invasive ventilation; SARI, severe acute respiratory infection; VM, Venturi Mask

The clinical outcomes of the enrolled patients are listed in Table 2. Compared to using HFCWO alone, patients using both BiPAP and HFCWO had shorter hospital stays (median hospital days: 21 vs. 27 days, *P* = 0.002), less frequent hospital stays of more than 23 days (41.1% vs. 61.1%, *P* = 0.002), and experienced a greater decline in sputum suction frequency (62.58% vs. 30.56%, *P* < 0.001). However, after excluding hospital days before the use of HFCWO, there was no statistical difference in hospital days between the two groups. There was also no statistical difference between the two groups in terms of oxygen demand post-HFCWO, tapering off oxygen post-HFCWO, respiratory failure, ICU admission, and hospital death.

**Table 2:** Clinical outcomes for enrolled patients who received HFCWO treatment

	No BiPAP use (*N* = 108)		BiPAP use (*N* = 163)		
	N	(IQR or %)	N	(IQR or %)	P-value^*^
Total hospital days, median (IQR)	27	30	21	16	0.002
Total hospital days > 23 days	66	61.1	67	41.1	0.002
Post HFCWO hospital days	15	(9, 24.5)	13	(9, 21)	0.285
Post HFCWO hospital stay > 15 days	50	46.3	61	37.4	0.184
Decrease sputum suction frequency	33	30.6	102	62.6	< 0.001
Post HFCWO oxygen use					0.538
Room air or nasal cannula	77	71.3	121	74.2	
VM FiO2 < 50%	13	12.0	13	8.0	
VM FiO2≥50% or MV	18	16.7	29	17.8	
Taper off oxygen post HFCWO	48	44.4	77	47.2	0.743
SARI CXR score decline	46	44.2	58	36.0	0.227
Respiratory failure	5	4.6	14	8.6	0.314
IMV	2	1.9	8	4.9	0.324
NIV	3	2.8	9	5.5	0.373
ICU admission	6	5.6	11	6.8	0.888
Hospital death	13	12.0	29	17.8	0.267

^*^Fisher exact test and Mann-Whiteny U test were used to calculate category variables and continuous variables, respectively

BiPAP, Bilevel positive airway pressure; HFCWO, high frequency chest wall oscillation; ICU, intensive care unit; IMV, invasive mechanical ventilation; IQR, interquartile range; MV, mechanical ventilation; NIV, non-invasive ventilation; SARI, severe acute respiratory infection; VM, Venturi Mask

**Table 3:** The univariable and multivariable logistic regression analysis of odds ratio of clinical outcomes for enrolled patients who received HFCWO treatment with or without bipap use

	Univariate			Multivariate^*^		
Clinical outcomes	OR	95% CI	p	OR	95% CI	p
Total hospital stay > 23 days	0.44	(0.27, 0.73)	0.001	0.58	(0.32, 1.04)	0.065
Post HFCWO hospital stay > 15 days	0.69	(0.42, 1.14)	0.147	1.01	(0.55, 1.87)	0.973
Decrease sputum suction frequency	3.80	(2.26, 6.38)	< 0.001	3.15	(1.53, 6.5)	0.002
Lower oxygen demand post HFCWO	1.12	(0.69, 1.82)	0.652	0.78	(0.46, 1.35)	0.383
Oxygen need post HFCWO	0.53	(0.32, 0.87)	0.013	0.46	(0.27, 0.79)	0.005
SARI CXR score decline	0.71	(0.43, 1.17)	0.182	0.72	(0.41, 1.25)	0.237
Respiratory failure	1.94	(0.68, 5.54)	0.218	2.12	(0.68, 6.60)	0.193
Post HFCWO with IMV	2.74	(0.57,13.13)	0.209	2.74	(0.57,13.13)	0.209
Post HFCWO with NIV	2.05	(0.54, 7.73)	0.292	1.57	(0.38, 6.57)	0.534
ICU admission	1.23	(0.44, 3.43)	0.692	1.64	(0.56, 4.77)	0.363
Hospital death	1.58	(0.78, 3.20)	0.203	2.04	(0.96, 4.32)	0.064

In univariable logistic regression analysis, patients using both BiPAP and HFCWO had a lower risk of total hospital days > 23 days and oxygen need post-HFCWO, and had higher odds of decreasing sputum suction frequency (Table 3). After multivariable analysis adjusting for confounders, patients using both BiPAP and HFCWO were associated with a lower risk of oxygen need post-HFCWO (OR: 0.55, 95% CI: 0.33–0.91, *P* = 0.021), and had a higher likelihood of decreased sputum suction frequency (OR: 2.91, 95% CI: 1.46–5.78, *P* = 0.002). Using both BiPAP and HFCWO was not associated with a decline in SARI CXR scores, lower total hospital days, lower post-HFCWO hospital days, a decreased risk of respiratory failure, ICU admission, and hospital death (Table 3).

Additionally, using HFCWO twice daily did not improve hospital days, sputum suction frequency, oxygen demand, respiratory failure, ICU admission, and hospital death, compared to using HFCWO once daily (data not shown).

After performing subgroup analyses, we found that patients with COPD, bronchiectasis, or those who received daily sputum suction more than 12 times did not experience clinical benefits, in terms of reduced sputum suction frequency or decreased oxygen demand, when using the combination of BiPAP and HFCWO (Supplementary Tables 1 to 3). In contrast, patients without COPD or bronchiectasis, and those who received daily sputum suction of 12 times or fewer, were associated with decreasing sputum suction frequency when using the combination of BiPAP and HFCWO.

## Discussion

After analyzing a retrospective cohort of 271 pneumonia patients who received HFCWO in our hospital, we found that the combination of BiPAP and HFCWO decreased the frequency of sputum suction, and reduced oxygen support levels more effectively than using HFCWO alone. However, adding BiPAP to HFCWO did not further decrease hospital stay, nor did it reduce the risk of respiratory failure, ICU admissions, or hospital mortality. Our results showed that the combination of BiPAP and HFCWO offer clinical benefits in facilitating sputum clearance and tapering oxygen support. However, this combination did not reduce the length of hospital stay, risk of respiratory failure, or hospital mortality.

In our study, we found that adding BiPAP to HFCWO can facilitate sputum clearance and improve oxygenation in patients with pneumonia. Pneumonia induces an inflammatory process in the respiratory system, increasing pulmonary vasodilatation and airway blocking with secretions, leading to ventilation-perfusion mismatch and hypoxemia [18]. BiPAP can delivers airflow into the lungs by creating a positive pressure gradient, preventing and treating pulmonary atelectasis, aiding sputum mobilization, and improving lung compliance [19]. On the other hand, HFCWO moves mucus from the peripheral lung to the central airway by producing high-frequency oscillations [20]. There may be a synergistic effect between BiPAP and HFCWO for lung expansion and secretion clearance, further improving oxygenation and decreasing the need for sputum suction in pneumonia patients.

We found that adding BiPAP to HFCWO did not further shorten hospital stays, nor did it lower the risk of respiratory failure, ICU admission, or mortality. To the best of our knowledge, there is no published research focusing on the combination of BiPAP and HFCWO for patients with pneumonia. In a previous randomized controlled trial with a small cohort conducted by Chen et al., intermittent positive pressure breathing (IPPB) might improve the weaning rate and reduce ventilator days for patients with prolonged mechanical ventilation [21]. A previous meta-analysis that included 13 studies assessed the effect of HFCWO on AECOPD patients and found that HFCWO may have the advantage of decreasing the length of hospital stays for AECOPD patients [15]. The difference in results between our study and previously published studies may be attributed to differences in the populations studied. Patients with bronchiectasis, COPD, or neuromuscular disorders may experience more significant benefits from ACT. Interestingly, in the subgroup analysis of our study, we found that patients without bronchiectasis and COPD derived greater benefit from the combination of HFCWO and BiPAP. A large-scale cohort study is warranted to confirm the benefit of combining BiPAP and HFCWO for this patient group.

Although our study found that the combination of BiPAP and HFCWO provides benefits in facilitating sputum clearance and reducing oxygen support, there are some limitations and potential complications associated with the use of BiPAP. The most well-recognized injuries include an increased risk of pneumothorax, pneumomediastinum, and subcutaneous emphysema [22]. Another issue is patient tolerance to the device, as the increase in airway pressure may cause discomfort, leading to difficulty in cooperating with BiPAP. Common interface-related side effects include discomfort, erythema, pressure ulcers, and skin rash [23]. Given these issues, it is crucial to closely monitor the patient’s condition during BiPAP intervention. BiPAP should be used with caution in the presence of structure lung disease, and patients at risk of pneumothorax, had unstable rib fractures, and facial trauma and deformities. Clinical healthcare providers should carefully assess the benefits and risks of using BiPAP for these patients.

This study had some limitations. First, the patients were retrospectively enrolled at a single medical center in Southern Taiwan, with a small sample size, and information bias cannot be excluded. Therefore, the results should be generalized with caution. Second, the decision to use BiPAP was based on clinical assessment. The characteristics between patients with or without BiPAP were different, so confounding by indication cannot be completely excluded. However, we included covariates related to patient demographics and pneumonia severity in the multivariable analysis, which likely adjusted for many confounders. Third, due to the retrospective design of our study, we were unable to use universal pneumonia severity indices, such as the Pneumonia Severity Index or CURB-65, for multivariate analysis to adjust for potential bias related to pneumonia severity. However, we adjusted for demographics, oxygen demand, SARI score, and suction frequency as surrogates for pneumonia severity.

## Conclusion

The combination of BiPAP and HFCWO demonstrated clinical benefits by decreasing the frequency of sputum suction and reducing oxygen support levels in pneumonia patients more effectively than HFCWO alone. However, it did not further decrease hospital stay post-HFCWO use, reduce the risk of respiratory failure, ICU admissions, or hospital mortality. Despite these limitations, the combined use of BiPAP and HFCWO may enhance sputum clearance and oxygen support management in pneumonia treatment. Further large-scale prospective cohort studies are necessary to confirm the efficacy of this combined management approach.

## Electronic supplementary material

Below is the link to the electronic supplementary material.

Supplementary Material 1

## Data Availability

All data are available from the corresponding author upon reasonable request.

## Abbreviations

ACT: airway clearance techniques
HFCWO: high-frequency chest wall oscillation
BiPAP: bi-level positive airway pressure
CPT: chest physiotherapy
CF: cystic fibrosis
NMD: neuromuscular disease
COPD: chronic obstructive pulmonary disease
BAL: bronchoscopic alveolar lavage
BMI: body mass index
CXR: chest X-ray
NIV: non-invasive ventilation
IMV: invasive mechanical ventilation
SARI: severe acute respiratory infection
IICP: increase intracranial pressure
IPAP: inspiratory positive airway pressure
EPAP: expiratory positive airway pressure
IPPB: intermittent positive pressure breathing
IQR: interquartile range
ORs: odds ratios (ORs)
CIs: confidence intervals

## Abbreviations

ACT: airway clearance techniques
BAL: bronchoscopic alveolar lavage
BiPAP: bilevel positive airway pressure
BMI: body mass index
CF: cystic fibrosis
Cis: confidence intervals
COPD: chronic obstructive pulmonary disease
CPT: chest physiotherapy
CXR: chest X-ray
EPAP: expiratory positive airway pressure
HFCWO: high-frequency chest wall oscillation
IICP: increase intracranial pressure
IMV: invasive mechanical ventilation
IPAP: inspiratory positive airway pressure
IPPB: intermittent positive pressure breathing
IQR: interquartile range
NIV: non-invasive ventilation
NMD: neuromuscular disease
Ors: odds ratios (ORs)
SARI: severe acute respiratory infection
