# Supplementary material for: Investigating the effectiveness of combining high-frequency chest wall oscillation with bilevel positive airway pressure in pneumonia patients: a retrospective cohort study
Source: BMC Pulm Med. 2025 May 3;25:214. doi: 10.1186/s12890-025-03685-y (PMC12048942; doi:10.1186/s12890-025-03685-y)
Supplement: Supplementary file 1 — Supplementary Material 1 [file 12890_2025_3685_MOESM1_ESM.docx]

| **Supplementary Table 1.** Subgroup analysis of patients with and without COPD by multivariable logistic regression | | | | | | | |
| --- | --- | --- | --- | --- | --- | --- | --- |
|  | With COPD | | |  | Without COPD | | |
| Clinical outcomes | OR | 95% CI | p |  | OR | 95% CI | p |
| Total hospital stay >23 days | 0.67 | (0.16, 2.89) | 0.592 |  | 0.38 | (0.21, 0.69) | 0.001 |
| Post HFCWO hospital stay>15 days | 3.18 | (0.60, 16.77) | 0.171 |  | 0.65 | (0.39, 1.23) | 0.184 |
| Decrease sputum suction frequency | 1.46 | (0.27, 7.83) | 0.659 |  | 3.29 | (1.54, 7.00) | 0.002 |
| Lower oxygen demand post HFCWO | – | – | – |  | 0.92 | (0.50, 1.50) | 0.413 |
| Oxygen need post HFCWO | * | * | 0.961 |  | 0.62 | (0.31, 1.24) | 0.179 |
| SARI CXR score decline | 0.18 | (0.04, 0.75) | 0.018 |  | 0.91 | (0.51, 1.64) | 0.751 |
| Respiratory failure | 4.19 | (0.32, 54.01) | 0.272 |  | 1.32 | (0.38, 4.57) | 0.662 |
| Post HFCWO with IMV | – | – | – |  | – | – | – |
| Post HFCWO with NIV | * | * | 0.956 |  | 1.68 | (0.32, 8.92) | 0.541 |
| ICU admission | * | * | 0.958 |  | 1.36 | (0.43, 4.35) | 0.601 |
| Hospital death | * | * | 0.938 |  | 1.35 | (0.62, 2.93) | 0.448 |
| * Indicates extremely high or low values.  – Multivariable analysis was not performed due to the absence of significant factors in the univariate analysis. | | | | | | | |

| **Supplementary Table 2.** Subgroup analysis of patients with and without bronchiectasis by multivariable logistic regression | | | | | | | |
| --- | --- | --- | --- | --- | --- | --- | --- |
|  | With bronchiectasis | | |  | Without bronchiectasis | | |
| Clinical outcomes | OR | 95% CI | p |  | OR | 95% CI | p |
| Total hospital stay >23 days | 0.77 | (0.11, 5.28) | 0.789 |  | 0.39 | (0.22, 0.70) | 0.001 |
| Post HFCWO hospital stay>15 days | 0.82 | (0.07, 9.25) | 0.870 |  | 0.81 | (0.45, 1.47) | 0.493 |
| Decrease sputum suction frequency | 15.16 | (0.27, 866,99) | 0.188 |  | 3.76 | (2.09, 6.75) | <0.001 |
| Lower oxygen demand post HFCWO | – | – | – |  | 0.81 | (0.45, 1.45) | 0.475 |
| Oxygen need post HFCWO | 0.45 | (0.03, 5.84) | 0.541 |  | 0.88 | (0.45, 1.73) | 0.711 |
| SARI CXR score decline | 2.22 | (0.37, 13.18) | 0.379 |  | 0.65 | (0.37, 1.16) | 0.147 |
| Respiratory failure | * | * | 0.959 |  | 1.52 | (0.48, 4.80) | 0.476 |
| Post HFCWO with IMV | – | – | – |  | – | – | – |
| Post HFCWO with NIV | * | * | 0.956 |  | 2.66 | (0.54, 13.11) | 0.228 |
| ICU admission | * | * | 0.958 |  | 1.63 | (0.56, 4.76) | 0.375 |
| Hospital death | * | * | 0.957 |  | 2.15 | (0.98, 4.74) | 0.056 |
| * Indicates extremely high or low values.  – Multivariable analysis was not performed due to the absence of significant factors in the univariate analysis. | | | | | | | |

| **Supplementary Table 3.** Subgroup Analysis of Patients by suction frequency by multivariable logistic regression | | | | | | | |
| --- | --- | --- | --- | --- | --- | --- | --- |
|  | Daily suction >12times | | |  | Daily suction ≤12times | | |
| Clinical outcomes | OR | 95% CI | p |  | OR | 95% CI | p |
| Total hospital stay >23 days | 0.40 | (0.17, 0.96) | 0.041 |  | 0.53 | (0.23, 1.19) | 0.121 |
| Post HFCWO hospital stay>15 days | 0.81 | (0.33, 2.01) | 0.648 |  | 0.93 | (0.42, 2.06) | 0.863 |
| Decrease sputum suction frequency | 2.29 | (0.88, 5.96) | 0.088 |  | 5.66 | (1.39, 23.09) | 0.016 |
| Lower oxygen demand post HFCWO | 0.97 | (0.39, 2.37) | 0.939 |  | 0.99 | (0.47, 2.09) | 0.970 |
| Oxygen need post HFCWO | 0.87 | (0.36, 2.07) | 0.751 |  | 0.87 | (0.32, 2.32) | 0.774 |
| SARI CXR score decline | 0.60 | (0.25, 1.43) | 0.248 |  | 0.68 | (0.34, 1.36) | 0.278 |
| Respiratory failure | 1.35 | (0.24, 7.73) | 0.729 |  | 2.32 | (0.57, 9.51) | 0.240 |
| Post HFCWO with IMV | 1.17 | (0.11, 12.63) | 0.896 |  | – | – | – |
| Post HFCWO with NIV | 0.88 | (0.08, 10.17) | 0.917 |  | 5.06 | (0.57, 44.57) | 0.144 |
| ICU admission | 1.42 | (0.15, 13.6) | 0.763 |  | 1.42 | (0.43, 4.70) | 0.565 |
| Hospital death | 2.61 | (0.70, 9.70) | 0.151 |  | 0.97 | (0.38, 2.50) | 0.949 |
| * Indicates extremely high or low values.  – Multivariable analysis was not performed due to the absence of significant factors in the univariate analysis. | | | | | | | |
